# Small airway dysfunction is associated to excessive bronchoconstriction in asthmatic patients

**DOI:** 10.1186/s12931-014-0086-1

**Published:** 2014-08-27

**Authors:** Veronica Alfieri, Marina Aiello, Roberta Pisi, Panagiota Tzani, Elisa Mariani, Emilio Marangio, Dario Olivieri, Gabriele Nicolini, Alfredo Chetta

**Affiliations:** Clinical & Experimental Medicine Department, University of Parma, Padiglione Rasori, via G. Rasori 10, 43125 Parma, Italy; Corporate Clinical Development, Chiesi Farmaceutici S.p.A, Parma, Italy

**Keywords:** Bronchial hyperresponsiveness, Small airways, Asthma

## Abstract

**Background:**

We investigated whether a relationship between small airways dysfunction and bronchial hyperresponsiveness (BHR), expressed both in terms of ease of airway narrowing and of excessive bronchoconstriction, could be demonstrated in asthma.

**Methods:**

63 (36 F; mean age 42 yr ± 14) stable, mild-to-moderate asthmatic patients (FEV_1_ 92% pred ±14; FEV_1_/FVC 75% ± 8) underwent the methacholine challenge test (MCT). The degree of BHR was expressed as PD_20_ (in μg) and as ∆FVC%. Peripheral airway resistance was measured pre- and post-MCT by impulse oscillometry system (IOS) and expressed as R5-R20 (in kPa sL^−1^).

**Results:**

All patients showed BHR to methacholine (PD_20_ < 1600 μg) with a PD_20_ geometric (95% CI) mean value of 181(132–249) μg and a ∆FVC% mean value of 13.6% ± 5.1, ranging 2.5 to 29.5%. 30 out of 63 patients had R5-R20 > 0.03 kPa sL^−1^ (>upper normal limit) and showed ∆FVC%, but not PD_20_ values significantly different from the 33 patients who had R5-R20 ≤ 0.03 kPa sL^−1^ (15.8% ± 4.6 vs 11.5% ± 4.8, p < 0.01 and 156(96–254) μg vs 207 (134–322) μg, p = 0.382). In addition, ∆FVC% values were significantly related to the corresponding pre- (r = 0.451, p < 0.001) and post-MCT (r = 0.376, p < 0.01) R5-R20 values.

**Conclusions:**

Our results show that in asthmatic patients, small airway dysfunction, as assessed by IOS, is strictly associated to BHR, expressed as excessive bronchoconstriction, but not as ease of airway narrowing.

## Introduction

Asthma is a chronic inflammatory disease affecting the entire tracheo-bronchial tree, from the proximal airways to the peripheral membranous bronchioles, the so-called small airways. The dysfunction of small airways may significantly influence the clinical manifestations and functional aspects of asthma [1]. Small airway obstruction was associated with poor disease control [2,3] and history of asthma exacerbations [3]. Importantly, small airways obstruction seems to significantly contribute to the degree of severity of bronchial hyperresponsiveness (BHR) [4,5], which is a functional hallmark of asthma [6], being a marker of worse disease outcome [7] and a risk factor for asthma development [8]. Notably, low values of the forced expiratory flow between 25% and 75% of vital capacity (FEF_25–75_) and of the forced expiratory flow at 50% of vital capacity (FEF_50_), considering both as measures of small airway patency, were strictly associated to BHR severity in children [4] and in adults [5] with asthma, respectively. In these studies, BHR was assessed as the provocative concentration [4] or dose [5] of methacholine causing a 20% fall (PC_20_ or PD_20_) in forced expiratory volume at 1^st^ second (FEV_1_).

**Figure 1:**
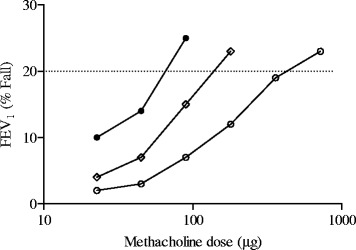
**Representative dose–response curves of three asthmatic patients to inhaled methacholine with different slope (%/μg) and PD**_**20**_ **(μg) values.** Patient A (closed circles) 0.248%/μg and 72 μg; patient B (open diamond) 0.112%/μg and 184 μg, patient C (open circles) 0.037%/μg and 625 μg.

It is of note that BHR, expressed as a dose–response curve to methacholine or histamine, may be characterized by two abnormalities consisting in a leftward shift of the dose–response curve (ease of airway narrowing) and in an upward displacement of the maximal response (excessive bronchoconstriction) (Figure 1). The PD_20_ (or PC_20_), a single point in the dose–response curve, reflects exclusively the ease of airway narrowing. Although both cross-sectional and longitudinal studies have shown a general relationship between PD_20_ (or PC_20_) values and severity of asthma, the within subject relationship was weaker [9]. On the other hand, the level of maximal response to the bronchoconstrictor stimuli reflects the propensity for airway closure and is presumably the most important physiopathological abnormality in asthma as long as it may determine fatal risk. Interestingly, excessive bronchoconstriction may be indirectly detected by measuring the fall in lung volume during bronchoprovocation testing.

Studies in humans [10] and in animal models [11,12] have shown that changes in lung volume and in parenchymal elastic load are related to the bronchoconstrictor response to methacholine. Notably, the maximal response of lung resistance to inhaled methacholine increases with decreasing lung volume and elastic recoil pressure [10–12]. Lung volume changes act by altering the forces of interdependence between airways and parenchyma that oppose airway smooth muscle contraction. Based on these findings, Gibbons et al. [13] proposed an indirect method for detection of excessive bronchoconstriction in patients with asthma by measuring the percentage fall in forced vital capacity (FVC) at PC_20_ (ΔFVC%) during bronchoprovocation testing. This index of airway closure was not related to the PC_20_ and was as reproducible as PC_20_ over a long period [13]. Furthermore, ΔFVC% correlated to asthma treatment in adult patients [13,14] and to the presence of symptoms in children with asthma [15].

The functional assessment of small airways is a challenging matter, given that the distal lung is relatively inaccessible for measurements. The ability of spirometry parameters to discriminate small airway obstruction is still debated [16]. On the other hand, the forced oscillation technique has been successfully used as a measure of the airway resistance heterogeneity and gas trapping, giving comparable results to the multiple breath nitrogen washout [17]. Furthermore, the impulse oscillometry system (IOS) has been increasingly used to measure both proximal and peripheral airway resistance in adults [2,3] and children [18,19] with asthma. The main advantage of this simple, noninvasive and sensitive technique [20] is that it does not require forced maneuvers, which may affect bronchial tone.

The aim of the present study was to ascertain whether a relationship between small airways dysfunction and severity of BHR may be demonstrated in patients with mild-to-moderate asthma. Small airway obstruction was assessed by means of IOS and BHR to methacholine was expressed both in terms of PD_20_, as a measure of ease of airway narrowing, and in terms of ΔFVC%, as a measure of excessive bronchoconstriction.

## Methods

### Subjects and study design

Adult patients (16 years of age and older, BMI ≤ 30 kg/m^2^) with asthma diagnosis according to the international guidelines [21], including current smokers, were eligible to take part in the study and were consecutively recruited from our Asthma Outpatient Clinic.

Between 9 and 12 a.m. on the same day, all patients underwent routine clinical history and physical examination. For each patient, BMI (kg/m^2^), atopy and asthma therapy were recorded. Asthma control was assessed using the Italian version of the Asthma Control Test [22]. Subsequently as part of their visit, patients underwent IOS before and after methacholine challenge testing (MCT). Patients were advised to omit inhaled bronchodilators 24 h before IOS and MCT.

The study was performed in accordance with the Good Clinical Practice guidelines recommended by the International Conference on Harmonization of Technical Requirements. The study was approved by the Ethics Committee for the Province of Parma (Italy) and all patients gave their informed consent.

### Impulse oscillometry

Impulse oscillometry was performed using the Jaeger MasterScreen-IOS (Carefusion Technologies, San Diego, CA, USA), following standard recommendations [20]. In short, patients were asked to wear a nose-clip and were seated during tidal breathing with their neck slightly extended and their lips sealed tightly around the mouthpiece, and while firmly supporting their cheeks with their hands. A minimum of three trials, each lasting 30 s, were performed and mean values were taken for each value.

Respiratory resistance at 5 and 20 Hz (R5 and R20, in kPa s l^−1^) were used as indices of total and proximal airway resistance, respectively and the fall in resistance from 5 to 20 Hz (R5-R20, in kPa s l^−1^) was considered as an index for the resistance of peripheral airways. Moreover, reactance at 5 Hz (X5, in kPa s l^−1^) was considered as a representative marker of peripheral airway abnormalities. Data are presented as raw data. An upper limit of normal for R5-R20 was chosen at 0.030 kPa s l^−1^, as previously reported [23]. Data obtained in our laboratory in 41 asymptomatic non-smoking subjects (24 F; age range 24–60 years; BMI range 18.0-29.7 kg/m^2^) without a history of lung disease and in whom spirometry results were within normal limits fell below these upper limits of normal. Our R5-R20 laboratory data were normally distributed with a mean ± SD value of 0.021 ± 0.029 kPa s l-1 (95% CI: 0.011-0.030 kPa s l^−1^).

### Lung function testing and methacholine challenge testing

Lung function was measured by a flow-sensing spirometer connected to a computer for data analysis (CPFS/D Spirometer, MedGraphics, St Paul, MN, USA) meeting the American Thoracic Society (ATS) standards. FVC, FEV_1_ and FEF_25–75_ were recorded and expressed as percent of predicted value. FEV_1_/FVC was also recorded and expressed as ratio.

Methacholine challenge testing was performed according to a standardized procedure. Each participant inhaled doubling increasing dose of methacholine (20–2400 μg), vaporized by a dosimeter with a driving pressure of 180 kPa generating an output of 10 μL/puff with particle size within the respirable range (<5 μM) (Dosimeter MB3; Mefar, Brescia, Italy). Patients underwent spirometry before and after each inhalation in order to record FEV_1_, FVC and FEF_25–75_. The test was stopped when either FEV_1_ fell by 20% or more from baseline measured after saline inhalation or 2400 μg methacholine cumulative dose was reached. PD_20_ in μg was calculated by linear interpolation of the dose–response curve and the values were log-transformed before analysis. A dose–response slope to methacholine (DRS,%/μg) was also calculated by least-squares linear regression analysis. The percentage fall in FVC (∆FVC%) at the PD_20_ relative to the baseline FVC after saline inhalation was also calculated using log-linear interpolation [13].

### Statistical analysis

The distribution of variables was assessed by means of Kolmogorov-Smirnov Goodness-of-Fit test. Variables are expressed as mean ± SD, unless otherwise specified. Unpaired and paired *t*-test, Mann Whitney test and Pearson χ^2^ test were used for comparisons, when appropriate. To examine relationships between measures Pearson’s correlation coefficient (*r*) and Spearman rank order correlation coefficient (*r*_*s*_) were used, when appropriate. The receiver operating characteristic (ROC) curve method [24] was used to plot the true positive rate (sensitivity) in function of the false positive rate (100-specificity), for different cut-off points of ∆FVC% with respect to R5-R20 > 0.030 kPa s l^−1^, as threshold value. A *p* value ≤ 0.05 was considered as significant.

## Results

We consecutively enrolled 71 patients (43 F, age range 16–74 yr) with asthma severity ranging from mild to severe. Eight patients were excluded due to a BMI ≥ 30 kg/m^2^. The 63 included patients showed FEV_1_ and FEV_1_/FVC values ranging respectively from 65 to 132% of predicted value and from 58 to 96%. Twenty-one out of 63 asthmatic patients were on ICS (500–1000 mcg/day of beclomethasone dipropionate or equivalent) plus long acting beta2-agonists, 9 patients were on ICS alone (500 mcg/day of beclomethasone dipropionate or equivalent) and the remaining 33 patients controlled their symptoms with inhaled salbutamol prn. Fifty-two patients were atopics (82%). Forty-nine out of 63 patients (78%) had well controlled asthma (ACT > 20). In all patients, the ACT median score was 24, ranging from 13 to 25.

**Figure 2:**
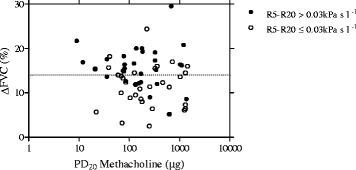
**Relationship between ∆FVC% and PD**_**20**_ **methacholine in 63 asthmatic patients with R5-R20 ≤ 0.03 kPa s l** ^**−1**^ **(** ***open circles*** **) and with R5-R20 > 0.03 kPa s l** ^**−1**^ **(** ***closed circles*** **).** The interrupted line represents the median value of ∆FVC%, corresponding to 14%. Twenty-one out of 30 patients with R5-R20 > 0.030 kPa s l^−1^ and 10 out 33 patients with R5-R20 ≤ 0.030 kPa s l^−1^ had a ∆FVC% value higher than the median value of ∆FVC% of the entire group of patients (χ^2^ = 7.222, p = 0.007).

**Figure 3:**
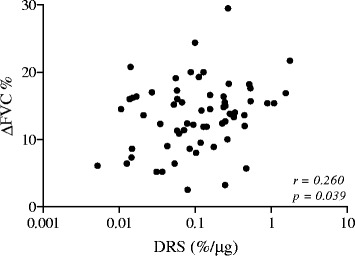
**Relationship between ∆FVC% and DRS in 63 asthmatic patients.**

**Figure 4:**
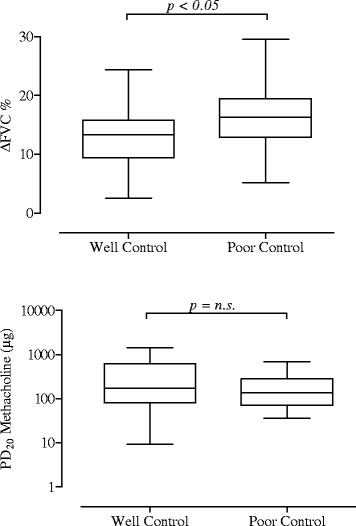
**Mean, standard deviation and range values of ∆FVC% (** ***upper panel*** **) and PD**_**20**_ **methacholine (** ***lower panel*** **) in 49 well controlled and 14 poorly controlled asthmatic patients.**

All patients showed BHR to MCT (PD_20_ < 1600 mcg) with a PD_20_ geometric (95% CI) mean value of 181 (132–249) μg, a DRS geometric (95% CI) mean value of 0.103 (0.074-0.143)%/μg and with a ∆FVC% mean and median value of 13.5% ± 5 and 14.0% respectively, ranging from 2.5 to 29.5% (Table 1). In all patients, there was no relationship between ∆FVC% and PD_20_ (r = −0.132; p = 0.303) (Figure 2). In contrast ∆FVC% and DRS values were weakly but significantly and positively related (r = 0.260; p = 0.039) (Figure 3). A significant difference was found in ∆FVC (16.1% ± 5.8 vs 12.8% ± 4.7; p = 0.037), but not in PD_20_ values [143(84–243) μg vs 200(132–286) μg; p = 0.431], when patients were subdivided in poorly controlled (ACT ≤ 19) and in well controlled (ACT > 19) (Figure 4). Pre- and post-MCT R5-R20 values were 0.057 kPa s l^−1^ ± 0.072 and 0.184 kPa s l^−1^ ± 0.148 (p < 0.001).

**Table 1:** **Characteristics of patients with asthma**

	**All patients (No. 63)**	**Patients with R5-R20 ≤ 0.03 kPa s l** ^**−1**^ **(No. 33)**	**Patients with R5-R20 > 0.03 kPa s l** ^**−1**^ **(No. 30)**
Age (years)	42 ± 14	38 ± 12	45 ± 15*
Sex (F/M)	36/27	13/20	23/7**
BMI (kg/m^2^)	24 ± 3	23 ± 2	25 ± 3
Atopy (Y/N)	52/11	31/2	21/9*
Smoking (Y/N)	12/51	9/24	4/26
ICS use (Y/N)	29/34	14/19	15/15
ICS dose (μg/day)^#^	701 ± 540	779 ± 598	628 ± 490
ACT > 20/ACT ≤ 19	49/14	29/4	20/10*
FEV_1_ (% of pred)	92 ± 14	94 ± 13	90 ± 15
FVC (% of pred)	99 ± 13	102 ± 12	97 ± 13
FEV_1_/SVC (%)	75 ± 8	75 ± 6	75 ± 9
FEF_25–75_ (% pred)	76 ± 25	77 ± 21	74 ± 29
R5-R20 (kPa s l^−1^)	0.057 ± 0.072	0.011 ± 0.014	0.108 ± 0.076**
X5 (kPa s l-1)	−0.134 ± 0.07	−0.098 ± 0.02	−0.172 ± 0.09**
PD_20_ (μg)	181 (132–249)	207 (134–322)	156 (96–254)
DRS (%/μg)	0.103 (0.074-0.143)	0.086 (0.054-0.135)	0.125 (0.076-0.205)
∆FVC%	13.6 ± 5.1	11.5 ± 4.8	15.8 ± 4.6**

Values are expressed as mean ± SD, ratio or geometric mean (Confidence Intervals).

^#^only in patients using ICS; *p < 0.05; **p < 0.01.

When patients were categorized by R5-R20 upper limit of normality [23], 30 out of 63 patients had R5-R20 > 0.030 kPa s l^−1^. The two groups of patients significantly differed in ∆FVC% value (p < 0.01), but not in PD_20_, in DRS and in spirometry values (Table 1). In addition, twenty-one out of 30 patients with R5-R20 > 0.030 kPa s l^−1^ and 10 out 33 patients with R5-R20 ≤ 0.030 kPa s l^−1^ had a ∆FVC% value higher than the median value of ∆FVC% of the entire group of patients (χ^2^ = 7.222, p = 0.007) (Figure 2). As compared to patients with ≤ 0.030 kPa s l^−1^, patients with R5-R20 > 0.030 kPa s l^−1^ were significantly older and also differed in gender, the vast majority being women. The ratio between the number of atopic and that of non atopic individuals and the ratio between the number of patients with well controlled asthma (ACT > 19) and that of patients with poorly controlled asthma (ACT ≤ 19) were significantly lower in patients with R5-R20 > 0.030 kPa s l^−1^, as compared to the patients with R5-R20 ≤ 0.030 kPa s l^1^ (2.3 vs 15.5; χ^2^ = 6.249, p = 0.012 and 2.0 vs 7.25; χ^2^ = 4.091, p = 0.043, respectively).

**Figure 5:**
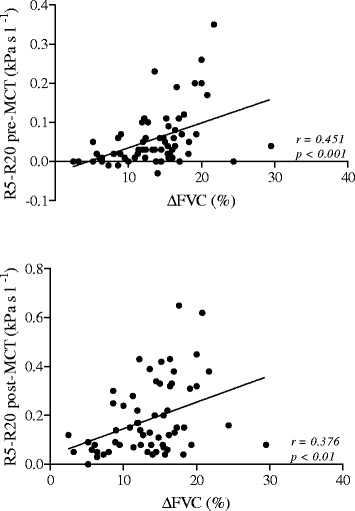
**Relationship between ∆FVC% and R5-R20 pre-MCT (** ***upper panel*** **) and R5-R20 post-MCT (** ***lower panel*** **) in 63 asthmatic patients.**

**Figure 6:**
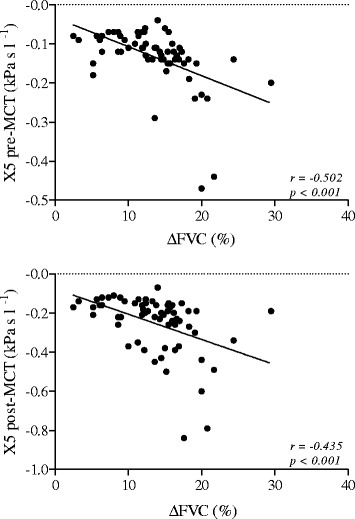
**Relationship between ∆FVC% and X5 pre-MCT (** ***upper panel*** **) and X5 post-MCT (** ***lower panel*** **) in 63 asthmatic patients.**

In all patients, ∆FVC% values were significantly related to the corresponding pre- (r = 0.451, p < 0.001) and post-MCT (r = 0.376, p < 0.01) R5-R20 (Figure 5) and pre- (r = −0.502, p < 0.001) and post-MCT (r = −0.435, p < 0.001) X5 (Figure 6) values, but not to the corresponding pre- (r = − 0.220, p = 0.082) and post-MCT (r = −0.117, p = 0.386) FEF_25–75_ values. In addition, according to the ROC curve method, the plot of the true positive rate in function of the false positive rate for different cut-off points of ∆FVC% with respect to R5-R20 > 0.03 kPa s l-1, as threshold value, showed a 0.758 (p < 0.01) area under curve value. The ∆FVC% cut-off point, which maximized sensitivity and specificity, was ≥ 14.5% (0.67 sensitivity and 0.76 specificity).

## Discussion

The main finding of this study is that a strict relationship between small airway dysfunction, as assessed by IOS, and bronchial hyperresponsiveness to methacholine is present in patients with mild to moderate asthma. The bronchial hyperresponsiveness can be demonstrated as ease of airway narrowing and as excessive bronchoconstriction expressed as PD_20_ and ∆FVC%, respectively. In the present study, those patients with increased peripheral airway resistance experienced a significantly higher ∆FVC% during the methacholine challenge than patients with normal peripheral airway resistance by showing, on the contrary, similar PD_20_ values. In addition, patients with small airway dysfunction were older, largely female and with a lower percentage of atopic and well-controlled individuals, as compared to the remaining ones.

Methacholine challenge testing is being used to assess hyperresponsiveness of the entire bronchial tree both in the clinical and research setting. However, whether deposition and effect of inhaled particles of the bronchoconstrictor agent may occur even in the small airways is still open to debate, by relying on different factors, such as inhalation manoeuver and particle size, which is in turn mainly determined by the nebulizer output. In patients with asthma, Cohen et al. [25] investigated whether small and large particle sizes of aerosolized adenosine monophosphate (AMP) lead to similar severity of airway hyperresponsiveness. They found that large-particle (9.9 μM) PC_20_ values were smaller than those of standard particles (3.7 μM), which in turn were smaller than small-particle (1.06 μM) PC_20_ values. These findings imply that the airway hyperresponsiveness degree is dependent on the particle size of the inhaled broncoconstrictor agent and, in this case, that small airways also do not show a similar severity of hyperresponsiveness compared to large airways. In our study, we used the MB3 Mefar Dosimeter, a largely used dosimeter, which at the recommended driving pressure of 180 kPa can assure an output with particle size within the respirable range (<5 μM) [26]. Accordingly, we may assume that in our asthmatic patients the aerosolized methacholine reached the lower airways by acting in this part of the tracheo-bronchial tree.

While a PD_20_ value is measurable in the vast majority of asthma patients during the methacholine challenge, a significant ∆FVC% value is not always detectable in asthmatic patients. In the present study, the ΔFVC values ranged from 2.5 to 29.5% with a mean value of 13.6% ± 5.1 and a normal frequency distribution. Our findings are very similar to those by Gibbons et al. [13] and Abisheganaden et al. [14], who previously reported a mean ∆FVC% of 13.2% ± 5.5 and 13.8% ± 4.8, respectively. Moreover, in line with these previous studies [13,14], we also found no relationship between ΔFVC% and PD_20_ values. Patients with the same value of PD_20_ may show different falls in FVC during methacholine challenge (Figure 2). Taken together, Gibbons et al. [13] Abisheganaden et al. [14] as well as our findings suggest that bronchial hyperresponsiveness *in vivo* is a composite functional disorder and that the mechanisms underlying excessive bronchoconstriction and ease of airway narrowing during the methacholine challenge are different. In our study, we also found that the ΔFVC% was significantly higher in patients with poorly controlled disease, as compared to well-controlled patients. These results are consistent with the findings of previous studies [13–15] that related ΔFVC% to the severity of asthma and considered it as a useful index in detecting the patients at risk for serious disease.

In the present study, we provide the first evidence that in asthmatic patients excessive bronchoconstriction expressed by ΔFVC% is strictly associated to small airway dysfunction, as assessed by IOS. As compared to patients with R5-R20 ≤ 0.030 kPa s l^−1^, patients with R5-R20 > 0.030 kPa s l^−1^ had a high likelihood to be associated to a ∆FVC% greater than 14.5% during a methacholine-induced bronchoconstriction. Moreover, in all patients we found a significant relationship between the baseline values both of the peripheral airway resistance and reactance, expressed respectively by R5-R20 and X5, and ∆FVC%. Three mechanical factors are called upon to explain the excessive airway narrowing in asthma: an increased contractility of airway smooth muscle induced by humoral mediators or abnormalities in neural control; a lack of a normal inhibiting factor which can prevent further shortening when the smooth muscle begins to shorten, so that the muscle never develops maximum force and degree of shortening; a decrease in elastic load, provided by cartilage and the surrounding parenchyma, so that it is easier to narrow an asthmatic bronchus as compared to a normal bronchus [27]. It is conceivable that the three above mechanisms may be amplified in the peripheral membranous bronchioles, which are without cartilage and, in asthmatic patients, may be the site of intense and extensive processes of inflammation [28–30] and remodeling [31,32]. Thus, inflammatory and structural changes may result in destabilization of these airways, which may become in turn prone to excessive bronchoconstriction. When directly measured *in vivo*, the small airway reactivity to histamine was significantly enhanced in asthmatic patients relative to normal controls with Isoproterenol being able to completely reverse the increase in peripheral airway resistance in the latter, but not in the former [33].

In line with previous studies, which showed a relationship between small airway dysfunction, as assessed by IOS, and uncontrolled disease, both in adults [2,3] and in children [34,35] with asthma, our group of patients with increased values of peripheral airway resistance had a greater proportion of poorly controlled asthma. Interestingly, these patients did not differ from patients with normal values of peripheral airway resistance in terms of FEV_1_ and FEV_1_/SVC. This is not surprising since patients with severe asthma may show similar airflow obstruction, but significantly increased air trapping, as compared to patients with non severe asthma [36]. Moreover, in our study patients with increased values of peripheral airway resistance were older, largely female and with a lower percentage of atopic individuals, as compared to the patients with normal values of peripheral airway resistance. Whether, age gender or atopy may directly affect the extent of small airway involvement in asthma has up to now not been deeply investigated. In a small group of asthmatic patients, males and females showed different small airways impairment [37]. Interestingly, males exhibited small airway involvement by attenuated small airway patency and females by small airway inflammation [37]. Air trapping, older age, female gender and less atopy were also significantly associated to the severe asthma phenotype [38].

## Conclusion

The present study shows that mild-to-moderate asthmatic patients with small airway dysfunction, as assessed by IOS, have excessive bronchoconstriction during the methacholine challenge, which is an important index of disease severity. Though association does not imply causality, the results of our study suggest a significant contribution of small airways in the pathophysiology of bronchial hyperresponsiveness. This fact may have implications for the treatment of asthmatic patients by increasing in importance the small-particle aerosols.

## Abbreviations

BHR: Bronchial hyperresponsiveness
DRS: Dose–response curve slope
FEV_1_: Forced expiratory volume at 1st second
FVC: Forced vital capacity
∆FVC%: Percentage fall in FVC at PD_20_
IOS: Impulse oscillometry system
MCT: Methacholine challenge test
PD_20_: Dose of methacholine causing a 20% fall in FEV_1_
R5-R20: Fall in resistance from 5 to 20 Hz
